# From the Vitamin D Paradox to Precision Nutrition: Targeted Supplementation, Assay Pitfalls, and Clinical Decision-Making

**DOI:** 10.1007/s13668-026-00780-2

**Published:** 2026-07-03

**Authors:** Maria Dalamaga, Dimitrios Tsilingiris, Dimitra Petropoulou, Rodopi Emfietzoglou, Maria Kypraiou, Dimitris C. Kounatidis, Natalia G. Vallianou, Spyridon Karras, Faidon Magkos, Irene Karampela

**Affiliations:** 1 Department of Biological Chemistry, Medical School, National and Kapodistrian University of Athens, Mikras Asias 75, Athens, 11527 Greece; 2 First Department of Internal Medicine, University Hospital of Alexandroupolis, Democritus University of Thrace, Alexandroupolis, Greece; 3 Assisting Nature Centre of Reproduction and Genetics, Thessaloniki, 57001 Greece; 4 Diabetes Center, First Propaedeutic Department of Internal Medicine, Medical School, National and Kapodistrian University of Athens, Laiko General Hospital, Athens, 11527 Greece; 5 First Department of Internal Medicine, Sismanogleio General Hospital, Athens, 15126 Greece; 6 Laboratory of Biological Chemistry, Medical School, Aristotle University, Thessaloniki, 55535 Greece; 7 Department of Nutrition, Exercise and Sports, University of Copenhagen, Rolighedsvej 26, Frederiksberg C, 1958 Denmark; 8 Second Department of Critical Care, Attikon General University Hospital, National and Kapodistrian University of Athens, Athens, 12462 Greece

**Keywords:** 25-hydroxyvitamin D, 25(OH)D, Assay standardization, Clinical decision-making, Precision nutrition, Randomized controlled trial, Supplementation, Vitamin D, Vitamin D deficiency

## Abstract

**Purpose of Review:**

Vitamin D is a pleiotropic secosteroid with established skeletal functions and proposed extraskeletal effects; however, its clinical application remains contentious. Despite extensive observational evidence linking low 25(OH)D concentrations to chronic diseases, randomized controlled trials (RCTs) have largely failed to confirm many of these associations. This review focuses on the practical clinical management of vitamin D, integrating determinants of vitamin D status, assay-related limitations, targeted testing, evidence-based supplementation, and decision-making within a precision-nutrition context.

**Recent Findings:**

The discordance between observational and interventional evidence is partly explained by the inclusion of vitamin D–replete populations in major trials, background supplementation, non-linear dose–response relationships, and assay variability. For skeletal outcomes, clinically meaningful effects are mainly observed in deficiency, whereas selected benefits have been reported in specific populations, including mortality reduction in adults aged ≥ 75 years, a modest reduction in cancer mortality, and reduced progression to diabetes in adults with high-risk prediabetes. The 2024 Endocrine Society guideline recommends empiric supplementation for selected groups and discourages routine screening in healthy adults. Daily or weekly dosing is preferred to intermittent high-dose regimens, which may increase falls and fractures. Standardization initiatives have improved 25(OH)D measurement accuracy, but inter-method variability persists, complicating thresholds and cross-study comparisons. Genetic polymorphisms in *GC*,* CYP2R1*,* CYP24A1*, and *VDR* contribute to variability in supplementation response, although precision-nutrition approaches remain investigational.

**Summary:**

Vitamin D should be viewed as a context-dependent, threshold-driven nutrient rather than a universal preventive therapy. Clinically, priority should be given to preventing and correcting severe deficiency (< 12 ng/mL [< 30 nmol/L]), a threshold below which adverse skeletal outcomes are well documented. Supplementation should target evidence-based indications, and laboratory testing should be reserved for situations in which results inform management. Bridging evidence and practice requires trials in deficient populations, improved assay harmonization, and integration of individualized risk factors into clinical decision-making.

## Introduction

Vitamin D is a pleiotropic secosteroid with hormone-like properties, exhibiting well-established roles in calcium homeostasis and skeletal integrity, alongside proposed functions in immune modulation, inflammation, cellular differentiation, and metabolic regulation [1–4]. Despite extensive literature, its clinical application beyond bone health remains debated. Over the past decades, observational studies have reported associations between low serum 25-hydroxyvitamin D (25(OH)D) concentrations and diverse chronic diseases, including cardiovascular, metabolic, autoimmune, infectious, and neoplastic conditions [1]. However, while most of these associations have not been consistently confirmed in randomized controlled trials (RCTs) and Mendelian randomization (MR) studies, notable exceptions include an association between genetically lower 25(OH)D and multiple sclerosis risk in MR studies, a modest reduction in cancer mortality in meta-analyses of RCTs, and mortality reduction in older adults in RCT-based guideline analyses, leading to ongoing debate regarding causality for many proposed extraskeletal effects and contributing to heterogeneity in clinical recommendations [5].

This discordance has translated into substantial variability in clinical practice. Key uncertainties include the definition of vitamin D deficiency and sufficiency, indications for laboratory testing, interpretation of circulating biomarkers in different clinical contexts, and optimal supplementation strategies. Analytical variability between assays, non-linear dose–response relationships, and interindividual differences in vitamin D metabolism, including genetic polymorphisms in vitamin D receptor (*VDR)*,* CYP2R1*,* CYP24A1*, and *GC* genes, further complicate evidence translation [6]. Consequently, clinicians face conflicting guidelines and unclear thresholds, increasing risks of both under-treatment and over-supplementation. Notably, the 2024 Endocrine Society (ES) guidelines no longer endorse the previous 30 ng/mL (75 nmol/L) target or specific thresholds for defining severe deficiency, deficiency, and sufficiency, reflecting the evolving understanding that outcome-specific benefits have not been established in clinical trials [5].

Moreover, vitamin D differs fundamentally from pharmacological agents, as its biological effects are highly context-dependent, with clinically meaningful supplementation benefits primarily observed in states of deficiency [5–7]. This concept challenges traditional trial designs and necessitates a more nuanced, individualized approach. Large RCTs in predominantly vitamin D-replete populations (e.g. VITamin D and OmegA-3 TriaL-VITAL and D-Health) have shown minimal benefit for most non-skeletal outcomes, whereas consistent evidence supports a role of vitamin D status in multiple sclerosis risk (from MR studies), a modest reduction in cancer mortality (from RCT meta-analyses), and all-cause mortality reduction in adults aged ≥ 75 years without evidence of a clear threshold effect, although data on effect modification by baseline 25(OH)D remain sparse [8]. Earlier evidence also suggested benefit for acute respiratory infections in deficient populations receiving daily or weekly supplementation; however, the most recent 2025 meta-analysis (40 studies; 61 589 participants) no longer demonstrates a statistically significant overall effect or clear effect modification by baseline vitamin D status [1, 7, 9, 10].

Accumulating evidence supports integrating biological, analytical, and clinical parameters to guide decision-making, moving toward a precision-nutrition model. Unlike recent reviews focused primarily on disease-specific health outcomes, the present review adopts a translational clinical-practice perspective, integrating determinants of vitamin D status, assay interpretation, targeted testing, supplementation strategies, guideline divergence, and precision-nutrition approaches. Therefore, this review provides a comprehensive, clinically oriented synthesis of vitamin D in clinical practice, addressing determinants of vitamin D status, analytical and methodological challenges in assessment, principles of clinical interpretation, evidence-based supplementation strategies, and practical decision-making approaches. Furthermore, it explores vitamin D as a model for precision nutrition and discusses public health strategies and ongoing controversies, thereby bridging the gap between evidence and real-world clinical application. Its novelty lies in integrating outcome evidence, assay-related pitfalls, divergent guideline thresholds, targeted supplementation, public-health strategies, and precision-nutrition concepts into a unified model for practical clinical decision-making.

## Literature Search

This article is a narrative review informed by a structured literature search. It was designed to provide a clinically oriented synthesis rather than a systematic review or meta-analysis. PubMed/MEDLINE, Scopus, and Web of Science were searched from database inception to March 2026 to identify evidence relevant to the clinical application of vitamin D. Search terms included combinations of “vitamin D,” “25-hydroxyvitamin D,” “assessment,” “measurement,” “analytical methods,” “supplementation,” “dose-response,” “clinical practice,” “guidelines,” “precision nutrition,” and “public health,” along with selected outcome-related terms when necessary to inform clinical decision-making.

Priority was given to high-level evidence, including systematic reviews, meta-analyses, RCTs, MR studies, and major clinical practice guidelines. Key methodological and analytical studies were also included to address laboratory variability, biomarker interpretation, and assay standardization. Landmark trials, such as VITAL and D-Health, and recent guideline updates were specifically considered because of their impact on clinical practice. Reference lists of selected articles were also reviewed to identify additional relevant publications.

Because this was not a systematic review, formal study screening by independent reviewers, risk-of-bias assessment, and quantitative evidence grading were not performed. Evidence was selected according to clinical relevance, methodological rigor, recency, and contribution to current controversies in the assessment and supplementation of vitamin D. Given the vast and heterogeneous nature of the vitamin D literature, this review does not aim to be exhaustive, but rather to provide a focused translational synthesis that supports practical, evidence-informed clinical decision-making.

## The Clinical Paradox of Vitamin D: Why Evidence and Practice Diverge

A striking discordance exists between observational studies and RCTs regarding vitamin D and health outcomes. While observational data consistently associate low serum 25(OH)D concentrations with increased risks of cardiovascular disease, cancer, diabetes, autoimmune conditions, and mortality, large RCTs have generally failed to confirm these associations [11–19]. This paradox has profound implications for clinical practice and guideline development.

**Fig. 1:**
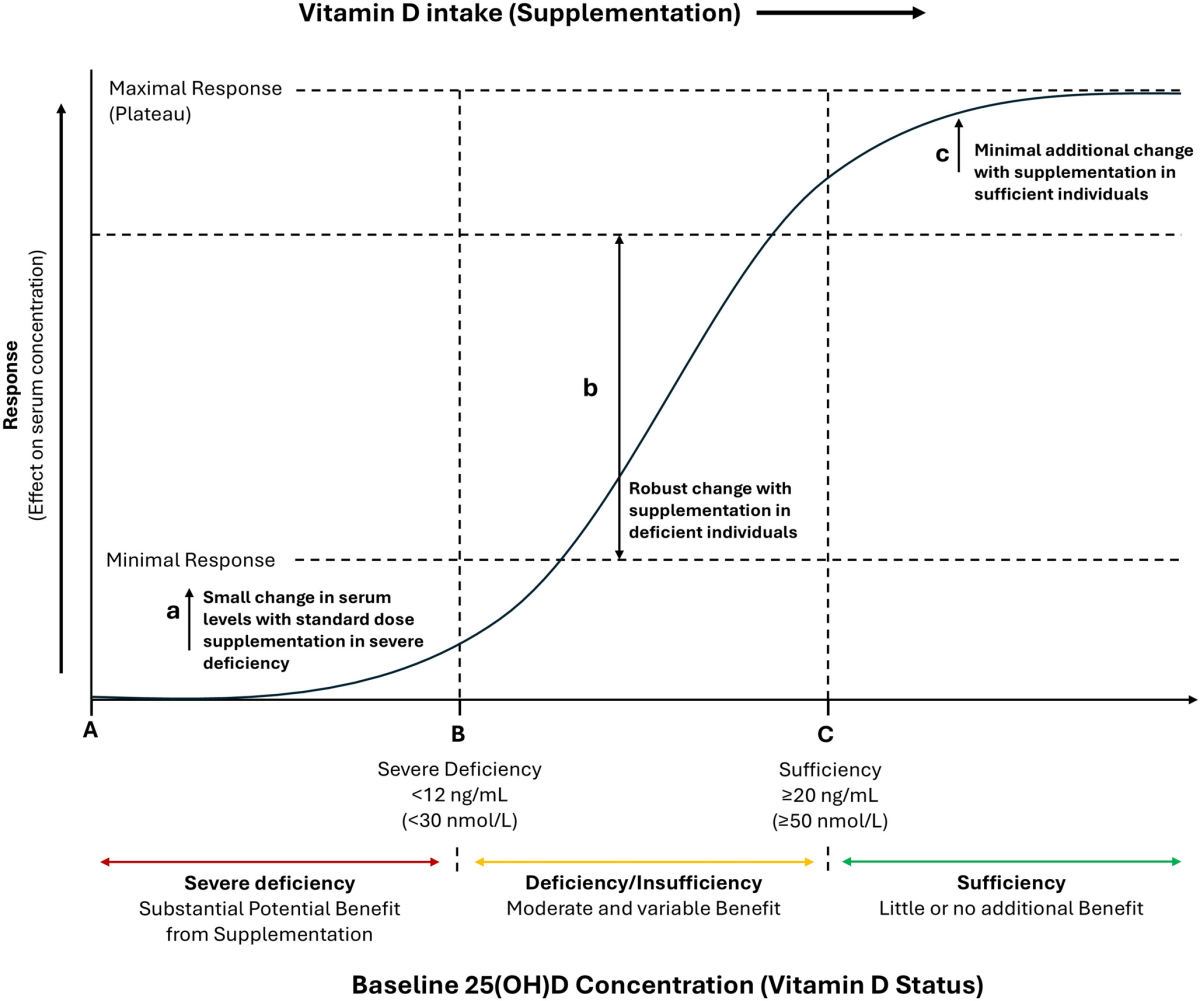
Intake–response relationship for vitamin D. The figure shows the non-linear (sigmoidal) relationship between vitamin D intake and physiological response according to baseline 25(OH)D status. The greatest benefit of supplementation occurs in severely deficient individuals (region A), whereas little or no additional benefit is observed in vitamin D–sufficient individuals (region C). On the other hand, a more pronounced increase of serum 25(OH)D concentration is observed with standard supplementation dose in individuals with deficiency/insufficiency (region B) whereas the dose-response plateaus in vitamin D–sufficient individuals (region C). This model helps explain why supplementation trials in largely replete populations often show null results for most outcomes; however, notable exceptions exist (e.g. cancer mortality and all-cause mortality in adults ≥ 75 years), where benefits have been observed even in replete populations, suggesting that this threshold model does not apply uniformly to all outcomes

The most compelling explanation for this discordance lies in the nutrient-response relationship. Unlike pharmacological agents, vitamin D exhibits a non-linear, sigmoidal dose–response curve, whereby individuals with deficient baseline levels show robust responses to supplementation, whereas those already replete demonstrate minimal benefit [20]. Critically, landmark trials such as VITAL and D-Health enrolled predominantly vitamin D-sufficient populations [1]. As the 2024 ES guidelines acknowledge, the absence of observed benefit in such trials does not exclude a biological effect, but rather reflects the adequacy of baseline vitamin D status for the outcomes studied [5]. Figure 1 illustrates the characteristic intake–response relationship observed for many nutrients, using vitamin D as a representative model, and highlights why identical supplementation doses may produce markedly different clinical effects depending on baseline vitamin D status.

Additional methodological features of vitamin D trials further contribute to null findings [21]. First, unlike pharmaceutical trials in which control participants receive no active agent, all participants in vitamin D trials are exposed through sunlight and diet, with many continuing background supplementation of up to 800 International Units (IU) daily. Second, most trials did not use baseline 25(OH)D as an eligibility criterion, preventing adequately powered subgroup analyses in deficient populations. Third, bolus dosing regimens (monthly or less frequent) may be less physiologically effective than daily administration [22]. A recent target trial emulation study using UK Biobank data showed that while null effects were expected in vitamin D-sufficient populations (mirroring VITAL and D-Health results), major mortality reductions (Hazard Ratios-HR 0.75–0.85) would be anticipated in populations restricted to those with deficiency or severe deficiency, supporting the need for RCTs specifically enrolling deficient populations [1]. Importantly, however, not all outcomes follow this deficiency-dependent pattern. Meta-analyses of RCTs have shown that daily vitamin D supplementation reduces cancer mortality by approximately 12% regardless of baseline vitamin D status, and the 2024 ES guideline found that vitamin D supplementation reduces all-cause mortality in adults aged ≥ 75 years across all baseline vitamin D levels [5, 23]. Similarly, in adults with high-risk prediabetes, vitamin D supplementation reduces progression to type 2 diabetes by approximately 15%, with benefits observed across a range of baseline vitamin D statuses, though potentially greater in those with 25(OH)D < 12 ng/mL (< 30 nmol/L) [24].

An alternative hypothesis posits that low 25(OH)D is a consequence rather than a cause of disease, a phenomenon consistent with reverse causation [22]. Systemic inflammation, reduced outdoor activity, and chronic disease states all lower circulating 25(OH)D concentrations. MR studies, designed to minimize confounding, have largely yielded null results for cardiovascular and metabolic outcomes, with the notable exception of multiple sclerosis, for which genetically lowered 25(OH)D has been consistently associated with increased disease risk [14]. However, MR studies have their own limitations, including assumptions about linear genetic effects that may not hold across the 25(OH)D distribution, and recent methodological refinements have cast doubt on earlier findings suggesting protective effects at low 25(OH)D concentrations [25].

This evidence landscape supports a nuanced, outcome-specific approach rather than a single paradigm. The 2024 ES guideline supports empiric supplementation for selected populations, including adults aged ≥ 75 years, pregnant women, children aged 1–18 years, and adults with high-risk prediabetes, although the certainty and nature of benefit vary by outcome [5, 11]. For other healthy adults aged 19–74 years without specific indications, the focus should be on preventing and correcting severe deficiency rather than on universal supplementation. The overarching priority should remain eliminating severe deficiency globally while directing empiric supplementation to evidence-based indications, a precision-nutrition approach that acknowledges the context-dependent biological effects of vitamin D [23, 24].

## Biological and Clinical Determinants of Vitamin D Status

Serum 25(OH)D concentrations show wide inter-individual variation influenced by both environmental and biological factors. Age, adiposity, diet, supplement use, seasonal and geographic variation in sunlight exposure are the main determinants, with ambient UV radiation and total vitamin D intake representing the strongest contributors [3, 4, 26]. Even among populations living in similar climates, differences in fortification policies and physical activity patterns contribute to substantial variability. Moreover, genetic polymorphisms affecting vitamin D synthesis, transport, and metabolism also exert a significant impact on circulating 25(OH)D levels [27].

Obesity represents a particularly important modifier of vitamin D homeostasis. Adults with obesity have a 3-fold higher prevalence of 25(OH)D levels below 20 ng/mL (< 50 nmol/L) compared to those without obesity [2, 28, 29]. This relationship is multifactorial, involving mechanisms such as volumetric dilution and sequestration in adipose tissue, reduced hepatic 25-hydroxylase (CYP2R1) activity, alterations in gut microbiota affecting absorption, and diminished outdoor activity [2, 28, 30]. Critically, the response to supplementation is also attenuated. Data from the VITAL trial showed that individuals with higher body mass index (BMI) achieved smaller increases in total, free, and bioavailable 25(OH)D despite identical supplementation doses [28].

Genome-wide association studies (GWAS) have identified polymorphisms in four key genes that collectively explain a substantial proportion of heritable variation in 25(OH)D: *GC* (encoding vitamin D-binding protein/VDBP), *CYP2R1*, *DHCR7/NADSYN1* (7-dehydrocholesterol reductase/NAD synthetase 1) (cutaneous synthesis), and *CYP24A1* (24-hydroxylase/catabolism) [31]. The rs10741657 polymorphism in *CYP2R1* is associated with an increased risk of vitamin D deficiency (Odds Ratio [OR] 1.42 under the dominant model [GG + AG vs. AA]; per-allele OR 1.09), while variants in *GC* and *VDR* modify the magnitude of response to supplementation, although the evidence for *GC* is mixed, with one study suggesting that the effect may be time-dependent and saturable after prolonged supplementation [32–34]. These gene–gene and gene–environment interactions have implications for personalized approaches to vitamin D management.

Skin pigmentation significantly affects cutaneous synthesis, with individuals with dark skin complexion having a 2- to 10-fold higher prevalence of deficiency depending on threshold definitions [35]. However, these estimates are based on total 25(OH)D levels, and controversy exists regarding whether this is the optimal measure of vitamin D status across racial groups, given differences in VDBP concentrations and genetic polymorphisms in *GC* [36]. Notably, the 2024 ES guideline uses “dark complexion” rather than race as the relevant biological variable, emphasizing that race is a social construct and an imprecise proxy for skin pigmentation [37]. Nevertheless, known risk factors, including age, BMI, latitude, season, and dietary intake, account for only 20 to 30% of the variation in 25(OH)D levels, underscoring the complexity of vitamin D homeostasis and the limitations of uniform population-based approaches [35].

## Assessment of Vitamin D Status: Analytical Challenges and Biomarker Evaluation

The circulating metabolite 25(OH)D is the preferred biomarker for assessing vitamin D status because it integrates cutaneous synthesis and dietary intake, reflects total body stores, and has a relatively long half-life of approximately 2–3 weeks, which refers to the plasma disappearance half-life measured by tracer kinetics. The functional half-life after cessation of supplementation or ultraviolet B (UVB) exposure may differ substantially. Its circulating concentration is about 1,000-fold higher than that of calcitriol, enabling reliable routine measurement, and it remains stable in serum and plasma under standard storage conditions [38]. However, total 25(OH)D does not fully capture the complexity of vitamin D metabolism, which has led to increasing interest in alternative or complementary biomarkers. Alternative biomarkers, such as the vitamin D metabolite ratio (VMR) or free/bioavailable 25(OH)D, may provide complementary information but are not yet validated for routine clinical practice [39, 40].

### Analytical Methods

The quantification of 25(OH)D is performed using chromatographic–mass spectrometric methods, such as high-performance liquid chromatography (HPLC) or liquid chromatography–tandem mass spectrometry (LC-MS/MS), or automated immunoassays (chemiluminescent, radioimmunoassays [RIA], or enzyme immunoassays). LC-MS/MS provides superior specificity, accuracy and traceability by distinguishing 25-hydroxyvitamin D₂ (25(OH)D₂) from 25-hydroxyvitamin D_3_ (25(OH)D₃) and resolving 3-epimer of 25-hydroxyvitamin D₃ (3-epi-25(OH)D₃), but is costly, technically demanding, requires skilled personnel, and shows inter-laboratory variability related to chromatographic separation, instrumentation and calibration [41]. Immunoassays, used in most routine laboratories (~ 80%), are simpler and faster but are susceptible to cross-reactivity and incomplete dissociation of 25(OH)D from vitamin D–binding protein (VDBP), leading to bias at low or high concentrations [42]. External quality-assessment programs (e.g. Vitamin D External Quality Assessment Scheme [DEQAS], Vitamin D Standardization Program [VDSP]) show inter-method variability of up to ± 20%, though the clinical impact may be greater. In one evaluation, only two of seven assays achieved an accuracy bias of < 5% against National Institute of Standards and Technology (NIST) reference materials, and the percentage of patients classified as deficient ranged from 1.5% to 14.3% depending on the assay used, with LC-MS/MS results generally closer to reference values but still affected by extraction and calibration differences, whereas immunoassays show method-dependent offsets, particularly in conditions with altered VDBP levels such as pregnancy, liver disease and nephrotic syndrome [42–45].

The VDSP coordinated by the NIH, the NIST, and the Centers for Disease Control and Prevention (CDC) established reference measurement procedures (RMPs) and certified reference materials, which substantially improved assay harmonization, although not all commercial methods meet recommended precision targets. Retrospective standardization of epidemiologic datasets has altered estimated deficiency prevalence, with the direction of change depending on the original assay. For example, in the National Health and Nutrition Examination Survey (NHANES) III (1988–1994), the percentage below 20 ng/mL (50 nmol/L) increased from 22% to 31%, whereas in the German Health Interview and Examination Survey for Children and Adolescents (KiGGS, 2003–2006) it decreased from 64% to 47%, highlighting the dependence of clinical decision limits on assay calibration [46]. Reporting concentrations in International System of Units (SI) units (nmol/L; 1 ng/mL = 2.5 nmol/L) is recommended, as mixed unit use remains a source of interpretative confusion; nevertheless, reporting both units is acceptable when conversion factors and reference ranges are clearly stated. While 25(OH)D remains the primary biomarker of vitamin D status, other metabolites, particularly the active hormone 1,25-dihydroxyvitamin D (calcitriol,1,25(OH)₂D), may provide complementary information in specific clinical contexts.

### Determination of 1,25(OH)_2_D

Compared to 25(OH)D, calcitriol circulates at approximately 1,000-fold lower concentrations (~ 50–160 pmol/L) and has a short half-life of about 5–8 h, being tightly regulated by parathyroid hormone (PTH), fibroblast growth factor 23 (FGF23), calcium, and phosphate [43]. Its laboratory determination is technically demanding. Although automated immunoassays are available and offer higher throughput, they show greater imprecision than LC–MS/MS and cannot reliably distinguish 1,25(OH)_2_D₂ from 1,25(OH)_2_D₃, a relevant limitation in settings in which vitamin D_2_ is commonly used [47]. The absence of reference measurement procedures and certified reference materials results in method-specific reference intervals [42]. Because serum 1,25(OH)₂D concentrations do not reliably reflect vitamin D stores, calcitriol measurement is not recommended for routine assessment of vitamin D deficiency, but is reserved for specific clinical indications, as summarized in Table 1 [48].

**Table 1:** Main clinical indications for calcitriol laboratory determination

Clinical indications	Rationale
Hypercalcemia of uncertain origin	Detects excess extrarenal 1α-hydroxylation (sarcoidosis, lymphoma)
Hypocalcemia with low PTH	Identifies 1α-hydroxylase or VDR defects
Chronic kidney disease (CKD)	Evaluates impaired renal synthesis (interpretation with PTH, FGF23)
Genetic or acquired rickets/osteomalacia	Differentiates calcipenic from phosphopenic forms
Granulomatous or malignant disorders	Monitors extrarenal production of calcitriol

CKD markedly affects vitamin D metabolism, with reduced renal 1α-hydroxylase activity leading to impaired calcitriol synthesis, and elevated FGF23 further suppressing CYP27B1 while stimulating CYP24A1-mediated catabolism. Accordingly, reliance on serum 25(OH)D alone may be misleading in CKD, and a comprehensive, but not routine, assessment incorporating PTH, calcium, phosphate, and FGF23 measurements is recommended [42].

### Clinical Relevance of other Vitamin D Forms and Metabolites

Beyond 25(OH)D and calcitriol, advances in analytical techniques have enabled the characterization of additional vitamin D metabolites that may refine the assessment of vitamin D metabolism. Advances in LC–MS/MS technology have transformed small-molecule clinical chemistry, enabling high-precision profiling of vitamin D metabolites. In circulation, approximately 85–90% of 25(OH)D is bound to VDBP, 10–15% to albumin, and less than 1% remains free; the albumin-bound and free fractions together constitute the bioavailable component, considered most relevant for cellular uptake [49]. While total, free, and bioavailable 25(OH)D show similar associations with health outcomes, no consistent incremental predictive value over total 25(OH)D has been demonstrated, except in conditions with altered VDBP concentrations such as pregnancy, nephrotic syndrome, liver failure, or acute inflammation [42]. The lack of standardized reference ranges further limits the routine clinical use of free and bioavailable 25(OH)D.

Beyond total 25(OH)D, downstream metabolites provide additional insight into vitamin D metabolism. The catabolic metabolite 24,25(OH)_2_D and the VMR (24,25(OH)_2_D/25(OH)D) reflect CYP24A1 activity and functional vitamin D sufficiency [43]. Low VMR values have been linked to CYP24A1 defects, functional vitamin D deficiency, elevated PTH, increased bone turnover, higher mortality, and reduced renal function in CKD, whereas elevated VMR may indicate excessive catabolism and reduced bioactive vitamin D [43]. Notably, the determination of 1,24,25(OH)₃D may assist in identifying dysregulated vitamin D metabolism in granulomatous disease or CYP24A1-related hypercalcemia [50].

Vitamin D epimers, specifically 3-epi-25(OH)D₃, arise from stereoisomeric modification and are particularly abundant in neonates and young infants, where they can account for up to 60% of total measured 25(OH)D [51, 52]. These epimers have lower affinity for the VDR and VDBP, and markedly reduced biological activity, but may interfere with 25(OH)D quantification in assays unable to resolve epimers, leading to overestimation of vitamin D status. A very recent DEQAS evaluation confirmed that 56% of LC-MS/MS laboratories did not effectively separate 3-epi-25(OH)D₃, and among immunoassays, the Roche Gen III showed 91% cross-reactivity [52]. Although their physiological role remains uncertain, limited evidence suggests low-potency effects on PTH regulation and calcium homeostasis [53].

Overall, although current guidelines do not recommend the routine determination of vitamin D metabolites, profiling 24,25(OH)_2_D, VMR, 1,24,25(OH)_3_D, and epimers may offer a more detailed evaluation of vitamin D metabolism, particularly in complex conditions such as CKD or genetic enzyme defects, though broader clinical adoption awaits assay standardization and validated reference ranges. Table 2 presents an overview of laboratory and clinical characteristics of vitamin D metabolites.

**Table 2:** Vitamin D metabolites: analytical and clinical characteristics

Metabolite	Role/Origin	Clinical biomarker utility	Determination methods	Clinical usefulness	Limitations
25-Hydroxyvitamin D [25(OH)D]	Main circulating storage form; produced in the liver (CYP2R1, CYP27A1).	-Accepted biomarker of vitamin D status for all populations -Reflects both dietary intake and cutaneous synthesis -half-life ~ 2–3 weeks, stable representation of vitamin D status -Concentrations in the nmol/L range -easier to measure reliably compared to other metabolites.	Immunoassays (CLIA, RIA, ELISA) or LC-MS/MS (reference method) -routinely measured -standardized assays available	- Primary biomarker for assessing vitamin D status in clinical and research setting -Correlates with outcomes - Widely used biomarker; clinical thresholds remain debated (e.g. <12 ng/mL [< 30 nmol/L] = severe deficiency; ≥20 ng/mL [≥ 50 nmol/L] = sufficiency), are evidence-based and reproducible across populations -Integrates into algorithms when combined with PTH, calcium, phosphate, and FGF23 to assess bone mineral metabolic disorders.	-Affected by assay bias, matrix effects, VDBP variations and epimers -Does not measure active hormone -Misleading in CKD or granulomatous disorders.
1,25-Dihydroxyvitamin D [1,25(OH)₂D, calcitriol]	Active hormonal form; produced mainly in kidneys (CYP27B1) and extra-renally in immune and epithelial tissues.	-Reflects activation capacity rather than stores; useful in CKD, granulomatous disease, and genetic hydroxylase defects -half-life ~ 5–8 h -Concentrations in the pmol/L range -↑ protein binding (99.9%)	-LC-MS/MS (reference) or automated immunoassays -requires sensitive assays	Diagnostic aid in hypercalcemia, sarcoidosis, hypoparathyroidism, pseudovitamin D-deficiency rickets, FGF23-related disorders	Unstable, low concentration, large biologic variation; poorly correlates with vitamin D status; interpretation requires PTH, Ca, phosphate.
24,25-Dihydroxyvitamin D [24,25(OH)₂D]	Catabolic product via CYP24A1; marker of 24-hydroxylase activity.	-Biomarker of catabolism and CYP24A1 activity -Used with 25(OH)D to calculate Vitamin D Metabolite Ratio (VMR = 24,25(OH)₂D/25(OH)D).	LC-MS/MS (multi-analyte targeted assay)	-Assesses vitamin D catabolism -Aids diagnosis of CYP24A1 deficiency (infantile hypercalcemia) and possibly fracture risk prediction	-Low concentrations; analytical complexity; influenced by renal function and supplementation dose. - Limited routine use
Vitamin D Metabolite Ratio (VMR)	-Functional marker integrating hydroxylation efficiency -catabolism	Marker of vitamin D catabolism and functional vitamin D status	Calculated via LC-MS/MS measurements of 25(OH)D and 24,25(OH)₂D	-Reflects metabolic activation vs. degradation - Discriminates functional deficiency, CYP24A1 defects -Potential index for bone turnover and CKD-MBD evaluation.	-Requires high-precision assays -Not standardized -Not validated for routine use.
Free/Bioavailable 25(OH)D	-Free 25(OH)D is the unbound fraction -bioavailable 25(OH)D includes free plus albumin-bound 25(OH)D -represent the biologically accessible pool	-May better reflect status in altered VDBP conditions (pregnancy, cirrhosis, critical illness, nephrotic syndrome) - May better reflect biologically available vitamin D in selected conditions	-Direct assay (ELISA, LC-MS/MS) -Calculated from VDBP and albumin levels	Supplementary biomarker in conditions with abnormal binding protein levels or gene polymorphisms	-No universal reference ranges -Limited added value in healthy individuals -High analytical complexity
3-epi-25(OH)D₃ and other epimers	C-3 epimeric forms, abundant in infants, formed via hepatic epimerase	-Detected in infants and pregnant women - May affect total 25(OH)D measurement	LC-MS/MS (epimer separation)	-Distinguishing true 25(OH)D₃ from epimers prevents overestimation of vitamin D status - Pediatric assessment -Research	-Unclear biological role - Interference in IAs
1,24,25(OH)₃D₃ and minor metabolites	-Further hydroxylated derivatives via CYP24A1 -Involved in feedback regulation	Research markers; explored for vitamin D toxicity and CKD progression	LC-MS/MS, usually in research laboratories	Potentially indicate excessive activation or catabolic balance	-Not yet validated for clinical use; very low serum levels; lack of reference intervals -Unclear clinical value

Abbreviations: 1,24,25(OH)₃D: 1,24,25-trihydroxyvitamin D; 1,25(OH)₂D: 1,25-dihydroxyvitamin D (calcitriol); 24,25(OH)₂D: 24,25-dihydroxyvitamin D; 25(OH)D: 25-hydroxyvitamin D; 3-epi-25(OH)D₃: 3-epimer of 25-hydroxyvitamin D₃; Ca: calcium; CKD: chronic kidney disease; CKD–MBD: chronic kidney disease–mineral and bone disorder; CLIA: chemiluminescent immunoassay; CYP24A1: cytochrome P450 24-hydroxylase; CYP27A1: cytochrome P450 27A1; CYP27B1: 1α-hydroxylase; CYP2R1: cytochrome P450 2R1 (vitamin D 25-hydroxylase); D₂: ergocalciferol; D₃: cholecalciferol; ELISA: enzyme-linked immunosorbent assay; FGF23: fibroblast growth factor- 23; IA: Immunoassay; LC–MS/MS: liquid chromatography–tandem mass spectrometry; PTH: parathyroid hormone; RIA: radioimmunoassay; VDBP: vitamin D–binding protein; VDSP: Vitamin D Standardization Program; VMR: vitamin D metabolite ratio (24,25(OH)₂D/25(OH)D)

## Clinical Interpretation of Vitamin D Measurements

Optimal circulating 25(OH)D thresholds remain debated, largely due to insufficient assay standardization, which contributes to variability in defining vitamin D sufficiency and underscores the need for harmonized methodologies. Reported target ranges differ because “optimal status” depends on the clinical context (skeletal versus extraskeletal outcomes) and the purpose of testing, whether for population-level screening or targeted assessment of high-risk individuals. Therefore, meaningful threshold determination requires integration of disease risk, clinical profile, and outcome-specific evidence [1].

The National Academy of Medicine (NAM), formerly the Institute of Medicine (IOM), and the Bone Health and Osteoporosis Foundation (BHOF), formerly the National Osteoporosis Foundation, define vitamin D status as “at risk for inadequacy” when serum 25(OH)D is < 12 ng/mL (< 30 nmol/L) and as “sufficient for nearly all” when it is ≥ 20 ng/mL (≥ 50 nmol/L). Some secondary sources simplify this as “deficiency” and “sufficiency,” although the NAM’s original terminology is more nuanced [35, 43, 54–59]. The prior (2011) ES guideline and some expert panels advocated higher targets, primarily to optimize bone and muscle health, although evidence for extraskeletal benefits remains inconsistent [6, 60]. However, the 2024 ES guideline no longer endorses a specific target threshold for sufficiency, citing a lack of outcome-specific clinical trial evidence. Concentrations less than 12 ng/mL (< 30 nmol/L) are clearly associated with rickets and osteomalacia, whereas concentrations between 20 and 50 ng/mL (50–125 nmol/L) are generally considered safe for skeletal health according to the NAM framework [1, 6]. Table 3 summarizes commonly used historical or guideline-specific categories of vitamin D status based on circulating 25(OH)D concentrations. These categories are presented as a pragmatic reference for clinical interpretation and should not be interpreted as universal treatment targets, particularly in light of the 2024 ES guideline, which does not endorse fixed 25(OH)D thresholds for sufficiency. Therefore, the interpretation of 25(OH)D concentrations should integrate the clinical context, the indication for testing, the assay used, and the outcome of interest rather than relying solely on a single universal cutoff. Finally, the timing of 25(OH)D measurement should account for seasonal variation, recent supplementation, and acute illness [4, 61]. Because serum levels typically reach their nadir at the end of winter, assessment during late winter or early spring may enhance detection of deficiency, when testing is clinically indicated [39, 62].

**Table 3:** Commonly used historical or guideline-specific categories of vitamin D status

Status*	Conventional units, ng/mL	SI units, nmol/L
Severe deficiency/high risk of deficiency-related skeletal disease	< 12	< 30
Deficiency or insufficiency according to several historical frameworks	12–20	30–50
Sufficiency (adequate for skeletal health according to NAM/IOM-type frameworks)	≥ 20	≥ 50
Optimal for high-risk or osteoporotic patients	30–50	75–125
Potential excess/increased toxicity risk	> 100	> 250

*These categories summarize commonly used historical or guideline-specific thresholds and should not be interpreted as universal targets. The 2024 ES guideline does not endorse a fixed 25(OH)D threshold for sufficiency because outcome-specific thresholds for benefit have not been established in clinical trials. Thresholds may vary according to guideline, population, assay method, and clinical outcome

Beyond threshold-based classification, the clinical interpretation of 25(OH)D is further limited by its biological and analytical constraints. Although 25(OH)D is the preferred measure of vitamin D status, it does not necessarily reflect tissue availability or intracellular activation [6]. Conversion to the active hormone 1,25(OH)₂D depends on PTH, FGF23, renal function, and inflammatory status, meaning that 25(OH)D may overestimate functional vitamin D sufficiency in CKD or inflammatory states and does not capture local extra-renal hydroxylation [63, 64]. Furthermore, as discussed in Sect. 5.3, approximately 85–90% of circulating 25(OH)D is bound to VDBP, with < 1% remaining free; clinical assays measure total 25(OH)D and do not distinguish bound from free fractions, potentially misrepresenting bioavailable vitamin D status in conditions with altered VDBP concentrations, such as pregnancy, liver disease, nephrotic syndrome, or acute inflammation [49, 65]. This limitation is particularly relevant in clinical settings characterized by altered protein binding or systemic inflammation. In addition, the poor correlation between 25(OH)D and the biologically active hormone is particularly evident in CKD and granulomatous disorders, where extrarenal 1α-hydroxylation or dysregulated catabolism may occur [38].

## Vitamin D Supplementation: Forms, Dosing, and Clinical Application

**Fig. 2:**
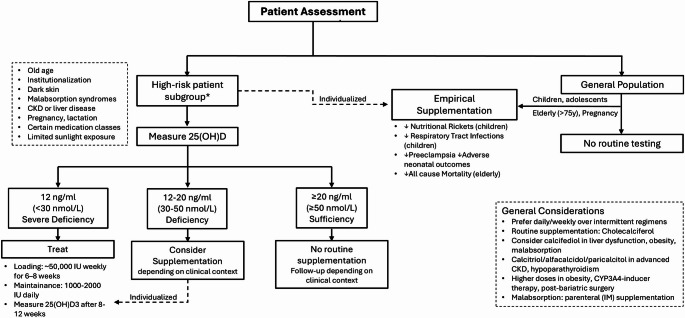
Clinical algorithm for Vitamin D screening and supplementation. Empiric supplementation without routine baseline 25(OH)D testing is suggested for children and adolescents, pregnant women, adults aged ≥ 75 years, and adults with high-risk prediabetes. In other individuals, clinical risk assessment should guide targeted 25(OH)D testing when results are expected to influence management. Daily cholecalciferol is preferred; weekly dosing is an acceptable alternative. Calcifediol may be appropriate in obesity, hepatic dysfunction, or malabsorption, whereas active vitamin D analogues are reserved for specific disorders, including advanced CKD, hypoparathyroidism, or impaired vitamin D activation

Figure 2 depicts a proposed practical algorithm for vitamin D assessment and supplementation.

### Forms and Indications

Several vitamin D compounds are available for clinical use, differing in potency, pharmacokinetics, and indications. The main forms include cholecalciferol (vitamin D₃), ergocalciferol (vitamin D₂), calcifediol (25(OH)D₃), and calcitriol. Cholecalciferol (vitamin D₃) is the native precursor requiring hepatic 25-hydroxylation, whereas calcifediol [25(OH)D₃] is the already hydroxylated form that bypasses this step and produces a faster, more predictable rise in circulating 25(OH)D concentrations.

Cholecalciferol is the preferred form for general supplementation owing to its longer half-life and greater potency than ergocalciferol. Vitamin D₃ raises serum 25(OH)D concentrations more efficiently than D₂, which is attributed to its higher affinity for VDBP and slower clearance [39, 66].

Calcifediol, which produces a faster and more linear increase in serum 25(OH)D, may be particularly useful in conditions such as hepatic dysfunction, obesity, or malabsorption, where hydroxylation or absorption of cholecalciferol may be impaired [39]. Calcitriol and synthetic analogues (e.g. alfacalcidol, paricalcitol) are reserved for advanced CKD, hypoparathyroidism and certain genetic disorders affecting 1α-hydroxylase activity [38, 67]. However, their narrow therapeutic window and risk of hypercalcemia and hyperphosphatemia limit their use for general supplementation [5].

### Doses and Factors Affecting Response

Current recommendations for vitamin D dosage vary by organization. The NAM (2011) recommends Dietary Reference Intakes (DRIs) of 600 IU for individuals aged 1–70 years and 800 IU for those over 70 years. The ES (2024) recommends following these DRIs for adults aged 19–74 years, while suggesting empiric supplementation (with a weighted average dose of approximately 900 IU/day in the supporting trials) for adults aged ≥ 75 years to reduce mortality risk [5, 55, 68]. The 2024 ES guideline does not specify a target 25(OH)D threshold, as outcome-specific thresholds for benefit have not been established in clinical trials [5]. The tolerable upper intake level (UL) is 4,000 IU/day for adults, though doses up to 10,000 IU/day are considered safe in the short term for deficiency correction [69].

A 2024 network meta-analysis of 35 RCTs (*n* = 58,937) found that daily administration of 800–1,000 IU vitamin D resulted in a lower risk of falls compared with placebo or no treatment (Relative Risk [RR] 0.85, 95% confidence interval [CI] 0.74–0.95), especially in individuals with baseline 25(OH)D ≤ 20 ng/mL (≤ 50 nmol/L), with a 31% reduction in this subgroup (RR 0.69, 95% CI 0.52–0.86), while intermittent dosing showed no preventive effect [70]. In the subgroup receiving daily dosing, the risk reduction was 22% (RR 0.78, 95% CI 0.64–0.92). The 2024 ES guideline reports that doses higher than 100,000 IU are associated with a trend toward increased fracture risk (incidence rate ratio [IRR] 1.23, 95% CI 0.81–1.61) compared with lower doses, and that longer dosing intervals (> 12 weeks) are linked to a higher fall risk (RR 1.08, 95% CI 1.03–1.14) [5].

The dose–response relationship is nonlinear and influenced by baseline 25(OH)D, BMI, genetics, age, absorption capacity and route of administration. Individuals with obesity or low VDBP levels often require two- to threefold higher doses to achieve equivalent increases in 25(OH)D. In vitamin D resistance, as seen with VDR mutations or hereditary vitamin D–resistant rickets, supraphysiologic doses of calcitriol or analogues, sometimes combined with calcium infusion, are necessary to overcome receptor insensitivity [39].

### Routes and Schedules of Administration

Vitamin D can be given orally or intramuscularly. Oral administration is preferred because of its better physiological absorption and safety profile, whereas parenteral routes are reserved for malabsorption syndromes or poor adherence.

Regarding frequency, daily or weekly dosing is superior to large intermittent (monthly or annual) boluses, which may transiently augment CYP24A1-mediated catabolism and cause supraphysiologic fluctuations in 25(OH)D, with transient elevations in FGF23 potentially increasing the risk of falls or bone resorption [71]. Nevertheless, the evidence regarding falls remains inconsistent, partly because reliable methods for fall assessment are lacking, as both self-reports and diary-based recordings have significant limitations. Daily supplementation provides more stable 25(OH)D concentrations and aligns better with physiological metabolism. However, intermittent dosing remains an option when compliance is difficult, provided that the total cumulative dose is appropriate [72].

### Duration of Treatment and Prevention

Correction of vitamin D deficiency typically requires 8–12 weeks of higher-dose therapy, followed by maintenance dosing. A common approach involves 50,000 IU weekly for 6–8 weeks, followed by transition to 1,000–2,000 IU daily. Maintenance therapy is usually lifelong in individuals with persistent risk factors (e.g. limited sunlight exposure, CKD, malabsorption) [5]. Periodic reevaluation of 25(OH)D levels may be considered in high-risk patients or after major health events that may affect vitamin D metabolism [73].

### Co-supplementation with Calcium, Magnesium, and Vitamin K

Calcium and vitamin D act synergistically in bone health. Co-supplementation is indicated in patients with osteoporosis, hypoparathyroidism, or in postmenopausal women with low dietary calcium intake [5, 74]. The typical calcium dose is 1,000–1,200 mg/day, tailored to dietary intake. However, calcium should not be routinely added to vitamin D in low-risk adults with adequate dietary intake, as combined use has been linked to a slight increase in nephrolithiasis risk and possibly to cardiovascular events [75].

Magnesium serves as a cofactor for vitamin D hydroxylases, and deficiency may blunt the response to supplementation [76]. Correcting magnesium deficiency may enhance 25(OH)D synthesis and PTH regulation. However, the relationship is complex, as one RCT showed that magnesium supplementation increased 25(OH)D₃ when baseline levels were near 30 ng/mL (75 nmol/L) but decreased it at higher baseline concentrations (~ 30–50 ng/mL [~ 75–125 nmol/L]), suggesting a regulatory rather than a simply additive effect [77]. Moreover, vitamin K_2_ (menaquinone) may complement vitamin D by activating osteocalcin and matrix Gla protein, promoting bone mineralization while preventing vascular calcification, though robust RCT data remain limited [78–80]. In particular, a meta-analysis of 8 RCTs found that combined vitamin K and D increased total bone mineral density (BMD) (effect size 0.316, 95% CI 0.031–0.601), but data on fracture reduction and vascular outcomes from large trials are lacking [81]. However, a 3-year RCT of vitamin K_2_ (375 µg/day MK-7) added to calcium and vitamin D in postmenopausal women with osteopenia showed no effect on BMD, bone turnover markers, or microarchitecture despite effective osteocalcin carboxylation, underscoring the need for caution in interpreting the meta-analytic findings [82].

### Adverse Effects and Toxicity

Vitamin D toxicity is rare but potentially serious, characterized by hypercalcemia, hypercalciuria, nephrocalcinosis and renal impairment. Chronic ingestion of more than 10,000 IU/day or serum 25(OH)D levels above 150 ng/mL (> 375 nmol/L) may precipitate toxicity, though the minimum level required to cause hypercalcemia is uncertain and may be lower in the presence of underlying hypercalcemic disorders or concurrent use of thiazide or lithium [83, 84]. Notably, large intermittent boluses, particularly ≥ 500,000 IU annually, have paradoxically increased the incidence of falls and fractures in elderly women [83, 85, 86]. Early symptoms of toxicity include polyuria, nausea, and muscle weakness, progressing to confusion or cardiac arrhythmias in severe cases. Prompt discontinuation of supplementation and hydration are usually sufficient, though corticosteroids or bisphosphonates may be required in persistent hypercalcemia [84].

### Monitoring After Treatment

Measurement of serum 25(OH)D may be considered 8–12 weeks after initiating or changing therapy in patients treated for confirmed deficiency, particularly in those with severe deficiency, malabsorption, CKD, obesity, or other conditions likely to impair response. In lower-risk patients receiving supplementation, reassessment before 6 months is usually unnecessary. Once stable maintenance therapy has been established, annual or seasonal reassessment may be sufficient in high-risk patients, whereas routine monitoring is not recommended for healthy adults receiving empiric supplementation within recommended intake ranges. Calcium, phosphate, and PTH should be monitored in patients receiving active vitamin D analogues, high-dose therapy, or treatment in the setting of CKD, hypoparathyroidism, granulomatous disease, or hypercalcemia risk [85, 87].

### Drug Interactions

Several medications may interfere with vitamin D absorption or metabolism, necessitating dose adjustments or enhanced monitoring. Bile acid sequestrants (cholestyramine) and lipase inhibitors (orlistat) reduce intestinal vitamin D absorption. Antiepileptic drugs (phenobarbital, phenytoin), rifampicin, and certain antiretroviral agents induce CYP3A4 and accelerate vitamin D catabolism, often requiring higher supplementation doses. More precisely, CYP3A4 induction by these agents promotes 4β-hydroxylation of 25(OH)D₃, an alternative catabolic pathway, rather than accelerating the canonical CYP24A1-mediated 24-hydroxylation [88]. Corticosteroids impair vitamin D metabolism and calcium absorption. Conversely, thiazide diuretics combined with calcium and vitamin D supplementation may precipitate hypercalcemia, particularly in elderly patients or those with renal impairment or hyperparathyroidism [60, 89, 90].

### Special Populations

#### Pregnancy

The 2024 ES suggests empiric vitamin D supplementation during pregnancy (600–5,000 IU/day range in the supporting trials), based on conditional guideline recommendations and trial evidence suggesting possible reductions in preeclampsia, intrauterine mortality, preterm birth, small-for-gestational-age birth, and neonatal mortality. Routine 25(OH)D testing is not recommended during pregnancy [5]. However, the estimated weighted average dose in the supporting clinical trials was approximately 2,500 IU/day, substantially higher than the 400–600 IU typically contained in prenatal vitamins [5].

#### Obesity and Bariatric Surgery

Vitamin D deficiency is highly prevalent in patients with obesity with up to 85% having 25(OH)D < 30 ng/mL (< 75 nmol/L) preoperatively. Individuals with obesity typically require 2–3 times higher doses to achieve equivalent increases in 25(OH)D levels. Following bariatric surgery, high-dose oral vitamin D₃ supplementation (≥ 2,000–3,000 IU/day, up to 6,000 IU/day) is recommended. In severe malabsorptive states, oral vitamin D₃ at 50,000 IU one to three times weekly or intramuscular administration may be preferable [91–96].

## Practical Approach to Vitamin D Management

Despite the high prevalence of hypovitaminosis D, universal population screening is not recommended, and a targeted approach is advised for individuals at increased risk. Routine screening may lead to unnecessary testing and overtreatment, whereas targeted testing improves cost-effectiveness and clinical relevance. The ES 2024 guidelines discourage routine testing in the general population due to insufficient evidence of outcome benefit [5]. The US Preventive Services Task Force (USPSTF) similarly concluded that the current evidence is insufficient to assess the balance of benefits and harms of screening for vitamin D deficiency in asymptomatic adults [35]. Testing may be justified in high-risk individuals, particularly when serum 25(OH)D concentrations fall below 12 ng/mL (< 30 nmol/L), a threshold associated with clinically significant adverse skeletal and health outcomes [39]. Table 4 outlines populations at increased risk of deficiency.

**Table 4:** Subjects at risk for vitamin D deficiency

High-risk group	Underlying mechanism
Older adults, institutionalized persons	↓ cutaneous synthesis, limited sun exposure
Individuals with dark skin	↑ melanin reduces UV-B absorption
Individuals with obesity	Sequestration of vitamin D in adipose tissue
Patients with malabsorption (e.g. celiac disease, Crohn’s disease, bariatric surgery)	↓ intestinal absorption
Parathyroid disorders	↓ Calcium absorption and no inhibitory control on PTH synthesis
Chronic kidney or liver disease	Impaired hydroxylation and metabolism
Pregnant or lactating women	↑ demand and plasma volume expansion
Infants of vitamin D-deficient mothers	↓ trans-placental transfer
Long-term glucocorticoid, anticonvulsant, antifungal, or rifampicin therapy	↑ catabolism via CYP induction
Covered populations (veiled, indoor workers)	Limited UV exposure

The 2024 ES guideline provides age-stratified recommendations: for nonpregnant adults aged 19–74 years, the panel suggests following the IOM Dietary Reference Intakes (600 IU/day for those aged 19–70 years; 800 IU/day for those older than 70 years), without routine supplementation beyond these levels or routine 25(OH)D testing; for adults aged 75 years and older, empiric supplementation is suggested due to a potential mortality benefit, with daily regimens preferred over intermittent high-dose administration. The guideline also suggests empiric supplementation for children and adolescents aged 1–18 years (to prevent nutritional rickets and potentially reduce respiratory infections) and for adults with high-risk prediabetes (to reduce progression to diabetes) [5]. These recommendations apply to generally healthy populations and do not supersede established indications for testing or treatment in individuals with metabolic bone disease, CKD, malabsorption, or other high-risk conditions [6].

In this context, measurement of 25(OH)D should be guided by specific clinical indications rather than routine screening. The determination of 25(OH)D is indicated in suspected osteomalacia, hyperparathyroidism, and CKD-related mineral and bone disorders, as well as in pediatric patients at high risk of deficiency or with growth delays. Monitoring intervals should depend on clinical indication and baseline risk. The Italian Association of Clinical Endocrinologists (AME) and the Italian Chapter of the American Association of Clinical Endocrinologists (AACE) position statement notes that in patients at risk of persistent low levels, retesting after 8–12 weeks may be appropriate, whereas in other patients retesting should not be performed before 6 months of supplementation [60]. Some expert recommendations support empiric vitamin D supplementation in the populations outlined in Table 4, regardless of baseline status, while reserving laboratory testing for situations in which results directly influence clinical management, such as differential diagnosis or monitoring of the response 3–6 months after therapy initiation [42].

Although the 2024 ES guideline does not recommend routine screening for vitamin D deficiency during pregnancy and suggests empiric supplementation regardless of 25(OH)D status [5], growing evidence supports optimization of maternal vitamin D status. Low maternal 25(OH)D has been associated with increased risks of preeclampsia, gestational diabetes, preterm delivery, and low birth weight, whereas supplementation has been associated with improved obstetric and neonatal outcomes and lower rates of infantile rickets [97–100]. Accordingly, maintaining vitamin D sufficiency during pregnancy and lactation may confer sustained skeletal and developmental benefits for both mother and child [101]. However, the updated 2024 Cochrane review by Palacios et al. substantially reduced the number of included studies (from 30 to 10) following trustworthiness assessments, resulting in downgraded evidence certainty for most pregnancy outcomes, and the evidence for effects on gestational diabetes, and other outcomes was very uncertain [102]. Thus, empiric vitamin D supplementation during pregnancy is reasonable and guideline-supported, whereas routine 25(OH)D screening remains unsupported in the absence of specific clinical indications; outcome certainty varies across pregnancy-related endpoints.

Overall, a targeted testing and supplementation strategy based on clinical context and baseline risk appears more appropriate than universal screening approaches.

## Vitamin D as a Model for Precision Nutrition

Precision nutrition aims to replace uniform dietary recommendations with strategies tailored to individual biological, genetic, and environmental characteristics [5]. However, at present, precision-nutrition approaches for vitamin D remain investigational and are not recommended for routine clinical use. Vitamin D exemplifies this paradigm, as its clinical effects are highly context-dependent, with meaningful benefits primarily observed in states of deficiency rather than uniformly across populations. Carlberg and colleagues have proposed the term “nutrigenomics” to describe this framework, positioning vitamin D as a prime example of how a single micronutrient can modulate the epigenome and transcriptome in a tissue- and individual-specific manner [103].

The concept of the “personal vitamin D response index” was proposed by Carlberg and Haq and captures this variability, distinguishing individuals into high, mid and low responders based on molecular markers such as changes in chromatin accessibility (assessed by formaldehyde-assisted isolation of regulatory elements followed by quantitative polymerase chain reaction, FAIRE-qPCR) and vitamin D–sensitive gene expression in peripheral blood mononuclear cells [104, 105]. This classification has been replicated across multiple cohorts. The VitDbol study (*n* = 35) segregated participants into 14 high, 11 mid, and 10 low responders, while the VitDPAS study (*n* = 45) identified 17 high, 19 mid, and 9 low responders, with 26 shared target genes confirmed across the VitDPAS and VitDHiD cohorts (total *n* = 70) at a false discovery rate of < 0.05 [104, 106]. This index reflects the efficiency of vitamin D signaling at the cellular level, which may not correlate directly with circulating 25(OH)D concentrations. Consequently, two individuals with identical serum levels may exhibit markedly different biological responses to supplementation.

This inter-individual variability, driven by genetic, metabolic, and environmental determinants discussed in previous sections, challenges the validity of universal dosing recommendations and fixed thresholds for sufficiency. A precision approach would integrate baseline vitamin D status, genetic risk scores (GRS), body composition, and clinical context to guide individualized supplementation strategies. A proof-of-concept study by Sallinen et al. demonstrated that a population-matched genetic risk score based on two single-nucleotide polymorphisms (SNPs, rs4588 in *GC* and rs10741657 in *CYP2R1*) could be used to tailor supplementation, reducing the difference in 25(OH)D between high- and low-risk genotype groups from 20.7 to 8.0 nmol/L (*p* = 0.0063) [107]. Similarly, gene–environment interaction analyses have shown that polygenic scores interact with vitamin D intake and UV exposure to influence 25(OH)D concentrations, particularly in European-ancestry populations [108]. Although routine genetic testing or molecular response profiling is not currently recommended for clinical vitamin D management, emerging pharmacogenomic data suggest that stratifying patients by response phenotype may eventually optimize dosing, reduce unnecessary testing, and improve clinical outcomes. Nevertheless, important limitations remain. The VIDARIS trial found that the effect of *GC* variants on 25(OH)D response was modest and disappeared after > 2 months of supplementation, suggesting that genetic influence may be time- and dose-dependent and saturable; and overall, known genetic variants explain only a small proportion of supplementation response variability [34, 109].

Therefore, vitamin D serves as a prototype model for developing precision nutrition strategies applicable to other micronutrients. Future directions include the development of validated algorithms incorporating multi-omic data, point-of-care genetic testing, and adaptive dosing protocols guided by real-time biomarker feedback [60]. However, it should be acknowledged that these approaches remain largely investigational. The personal vitamin D response index has been tested only in small cohorts of healthy individuals (*n* = 25–70), no clinical trial has yet demonstrated that molecular response-guided dosing improves hard clinical outcomes, and the cost-effectiveness and scalability of epigenomic or transcriptomic profiling for routine clinical use have not been established [104–106].

## Public Health Approaches: Fortification versus Supplementation

Food fortification with vitamin D represents an effective population-wide strategy to address widespread insufficiency, particularly in high-latitude regions with limited sunlight exposure. Key advantages include broad reach, low cost, and proven efficacy in increasing mean serum 25(OH)D concentrations across age groups without increasing the risk of toxicity. Meta-analyses indicate that fortification increases 25(OH)D by approximately 6–10 ng/mL (15–25 nmol/L) depending on the dose and population studied. Reported dose–response relationships are variable, with increases of roughly ~ 1–2 nmol/L per 1–2.5 µg/day (~ 40–100 IU/day) ingested in adults and ~ 2.5–7 nmol/L per 1–2.5 µg/day (~ 40–100 IU/day) in children [110–112]. These considerable differences may emerge due to the presence of non-linear dose-response effects [111]. Fortified foods such as milk, dairy products, margarine, cereals, and plant-based beverages provide a steady, physiological intake, reducing reliance on supplements and improving nutritional equity [113]. Experience from Finland shows that systematic fortification of fluid milk products and fat spreads, initiated in 2003 and doubled in 2010, increased mean 25(OH)D from 19.2 to 26 ng/mL (48 to 65 nmol/L) over 11 years, with 91% of supplement non-users who consumed fortified foods based on Finnish nutrition recommendations reaching ≥ 20 ng/mL (≥ 50 nmol/L) [114, 115]. In Canada, mandatory fortification of milk and margarine has contributed to a population mean 25(OH)D of 23.2 ng/mL (57.9 nmol/L), though 19% remain below 16 ng/mL (40 nmol/L), with marked disparities among racialized groups [116]. Therefore, mandatory or semi-mandatory fortification may reduce severe deficiency and seasonal variability [117]. Co-fortification with calcium may provide additional skeletal benefits, particularly in older adults and populations with low dietary calcium intake [113].

However, several limitations constrain universal implementation. Variability in dietary patterns, bioavailability, and limited access to fortified foods may reduce effectiveness in certain subpopulations, while voluntary fortification often results in inconsistent vitamin D content [118]. Modelling studies using NHANES data have shown that fortification disproportionately shifts the upper tail of the 25(OH)D distribution, potentially increasing the prevalence of concentrations above 50 ng/mL (> 125 nmol/L) from < 1% to ~ 8%, underscoring that fortification is “at best a blunt instrument” requiring careful calibration [119]. Regulatory concerns about over-fortification persist, particularly in settings with multiple vitamin D sources (diet, supplements, sunlight) [120]. The European Food Safety Authority **(**EFSA) has established a Tolerable Upper Intake Level (UL) of 100 µg (4,000 IU)/day for adults and adolescents aged 11–17 years, and 50 µg (2,000 IU)/day for children aged 1–10 years, and current European intake data suggest that populations are unlikely to exceed these limits except among regular users of food supplements containing high doses of vitamin D [121]. Supplementation, while effective for targeted correction, is limited by poor adherence, particularly among individuals with low socioeconomic status, and by the potential for overdosing with unsupervised use. Importantly, the long-term effects of chronic low-dose fortification on extraskeletal outcomes remain uncertain [122].

Europe has adopted a cautious approach to mandatory vitamin D fortification due to regulatory, cultural, and logistical factors. The EU’s centralized food legislation emphasizes consumer autonomy and safety, resulting in heterogeneous national policies rather than unified mandates [123]. Diverse dietary patterns, skepticism toward “mass medication,” and inconsistent labeling and upper intake thresholds further complicate implementation. Notably, 94–100% of Europeans aged ≥ 13 years fail to meet the reference intake of 10 µg/day (400 IU/day), underscoring the need for action [124]. Furthermore, only approximately 1.2% of prepackaged foods and drinks in Europe are voluntarily fortified with vitamin D, with margarine and plant-based drinks providing the majority of fortified vitamin D intake [124].

Nevertheless, expert groups such as the European Calcified Tissue Society (ECTS) increasingly support a coordinated European strategy combining moderate fortification, targeted supplementation of specific risk groups (infants and children up to 3 years, pregnant women, older persons, and non-Western immigrants), and nutritional education as a sustainable approach to improving population vitamin D status [120, 122, 123, 125]. Economic modelling suggests that wheat flour fortification is cost-saving, with costs offset by prevented cases of deficiency, while combined fortification and supplementation strategies are highly cost-effective [126]. Additionally, Niedermaier et al. projected that if all 34 European countries implemented adequate vitamin D food fortification, approximately 129,000 cancer deaths per year could be prevented, corresponding to approximately 9% of cancer deaths; however, this estimate is model-based and depends on the assumption that fortification-induced increases in 25(OH)D would yield cancer mortality reductions comparable to those observed in supplementation RCTs (~ 13%) [127].

## Challenges, Controversies and Divergent Guidelines in Vitamin D Practice

Accurate assessment of 25(OH)D remains challenging. Assay inconsistency represents a major and often underappreciated source of error, with systematic bias between immunoassays exceeding biological variability and undermining comparability across studies. Differences between immunoassays and LC–MS/MS, variable calibration to NIST standards, and inconsistent reporting units introduce misclassification bias, potentially distorting prevalence estimates and effect sizes [42, 44]. A recent multi-laboratory study evaluating 13 immunoassays and two LC-MS/MS methods found that slightly more than half met the desirable Joint Committee for Traceability in Laboratory Medicine (JCTLM) Task Force on Reference Measurement System Implementation measurement uncertainty threshold (≤ 10%), while four exceeded the minimum acceptable limit (≤ 15%), confirming that measurement uncertainty remains a major challenge even with current standardization efforts [128]. Without assay harmonization, meta-analytic pooling of data from heterogeneous laboratories introduces further noise. Furthermore, laboratory experts emphasize that the lack of global standardization and inconsistent measurement units (ng/mL vs. nmol/L) continue to cause confusion, thereby biasing thresholds for deficiency and sufficiency across populations and undermining comparability between guidelines [42].

In addition to analytical variability, population heterogeneity further complicates the interpretation of vitamin D status. Non-Hispanic Black individuals exhibit lower 25(OH)D levels yet paradoxically lower fracture rates than White individuals, raising questions about the universal applicability of current thresholds and about whether the same adequacy thresholds should apply to all racial groups [36, 129]. However, the 2024 ES guideline cautions that racial categories represent social rather than biological constructs, and that using race as a proxy for skin complexion is subject to ecological fallacy and will misclassify many individuals. Other factors such as social determinants of health may confound the relationship between self-identified race and vitamin D-related outcomes [5].

Guidelines differ markedly in defining deficiency and sufficiency. The 2024 ES guideline focuses on RCT-based evidence, recommending empiric supplementation for children and adolescents aged 1–18 years to prevent nutritional rickets and potentially reduce respiratory tract infections, for pregnant individuals to lower risks of preeclampsia and adverse neonatal outcomes, and for adults aged 75 years and older to reduce all-cause mortality. The guideline also suggests empiric supplementation for adults with high-risk prediabetes to reduce progression to type 2 diabetes. It discourages routine screening for 25(OH)D in healthy individuals, including those with obesity or dark complexion, and in those who are pregnant, and does not support high-dose vitamin D therapy or supplementation above the Dietary Reference Intake in nonpregnant healthy adults younger than 75 years [5]. In contrast, earlier ES guidelines (2011) advocated higher targets (≥ 30 ng/mL [≥ 75 nmol/L]) to optimize skeletal and extraskeletal outcomes [39, 130]. The NAM retains lower sufficiency thresholds (≥ 20 ng/mL [≥ 50 nmol/L]) based primarily on skeletal outcomes. These discrepancies reflect differing weights assigned to observational versus interventional evidence and divergent clinical versus public-health perspectives. Holick has critiqued the 2024 ES guideline for focusing exclusively on RCTs and ignoring association studies and other evidence supporting extraskeletal benefits, and for recommending that nonpregnant adults aged up to 75 follow only the IOM DRIs (600–800 IU/day), arguing that a preferred 25(OH)D range of 40–60 ng/mL (100–150 nmol/L), as recommended in the 2011 guidelines, better reflects the totality of evidence [131].

Importantly, the evidence base underlying these recommendations remains limited. Notably, no RCTs have been designed or powered to determine outcome-specific 25(OH)D thresholds, and trial durations are often insufficient to assess effects on chronic diseases with long latency periods, further limiting evidence-based threshold recommendations.

The dramatic increase in vitamin D testing, with Medicare reimbursement volumes increasing more than 80-fold from 2000 to 2010 in the USA, without established cost-effectiveness or evidence of improved clinical outcomes raises concerns about overdiagnosis and resource utilization [35, 132, 133]. The USPSTF (2021) specifically noted that screening may misclassify persons due to uncertainty about the cutoff for defining deficiency and the variability of available testing assays, potentially resulting in overdiagnosis (leading to nondeficient persons receiving unnecessary treatment) or underdiagnosis [35]. Furthermore, population-level screening may worsen health equity, as it requires resources not universally accessible, whereas empiric supplementation in high-risk groups may represent a more equitable approach [5].

In response to these challenges, emerging approaches emphasize a shift toward individualized assessment. Some viewpoints advocate incorporating genetics, BMI, comorbidities, and lifestyle factors rather than relying on uniform population targets [134]. Others support integrated biomarker strategies combining 25(OH)D with calcium, PTH and FGF23 to better contextualize bone–mineral metabolism [42]. Herrmann et al. showed that a “low vitamin D metabolite profile”, defined by 24,25(OH)₂D < 1.2 ng/mL (< 3 nmol/L) and VMR < 4%, identified individuals with accelerated bone metabolism and higher all-cause mortality more accurately than a fixed 25(OH)D cutoff of 20 ng/mL (50 nmol/L), suggesting that functional metabolite profiling may refine individualized assessment [40]. Finally, although total, free and bioavailable 25(OH)D show similar inverse associations with mortality, current evidence does not support additional predictive value of alternative fractions over total 25(OH)D in the general population [135, 136]. However, in patients with coronary artery disease, Yu et al. found that bioavailable and free 25(OH)D, but not total 25(OH)D, were independently associated with all-cause and cardiovascular mortality, suggesting that the relative value of these fractions may be context-dependent.

The limitations of this review should be acknowledged. First, this is a narrative review informed by a structured search strategy rather than a systematic review; therefore, formal risk-of-bias assessment and quantitative evidence grading were not performed. Second, vitamin D studies are heterogeneous with respect to populations, baseline 25(OH)D status, dosing regimens, assay methods, and outcomes, limiting direct comparisons across studies. Third, many trials and meta-analyses were not designed to define outcome-specific 25(OH)D thresholds. Finally, precision-nutrition approaches remain investigational and have not yet been validated for routine clinical decision-making.

## Conclusion

**Fig. 3:**
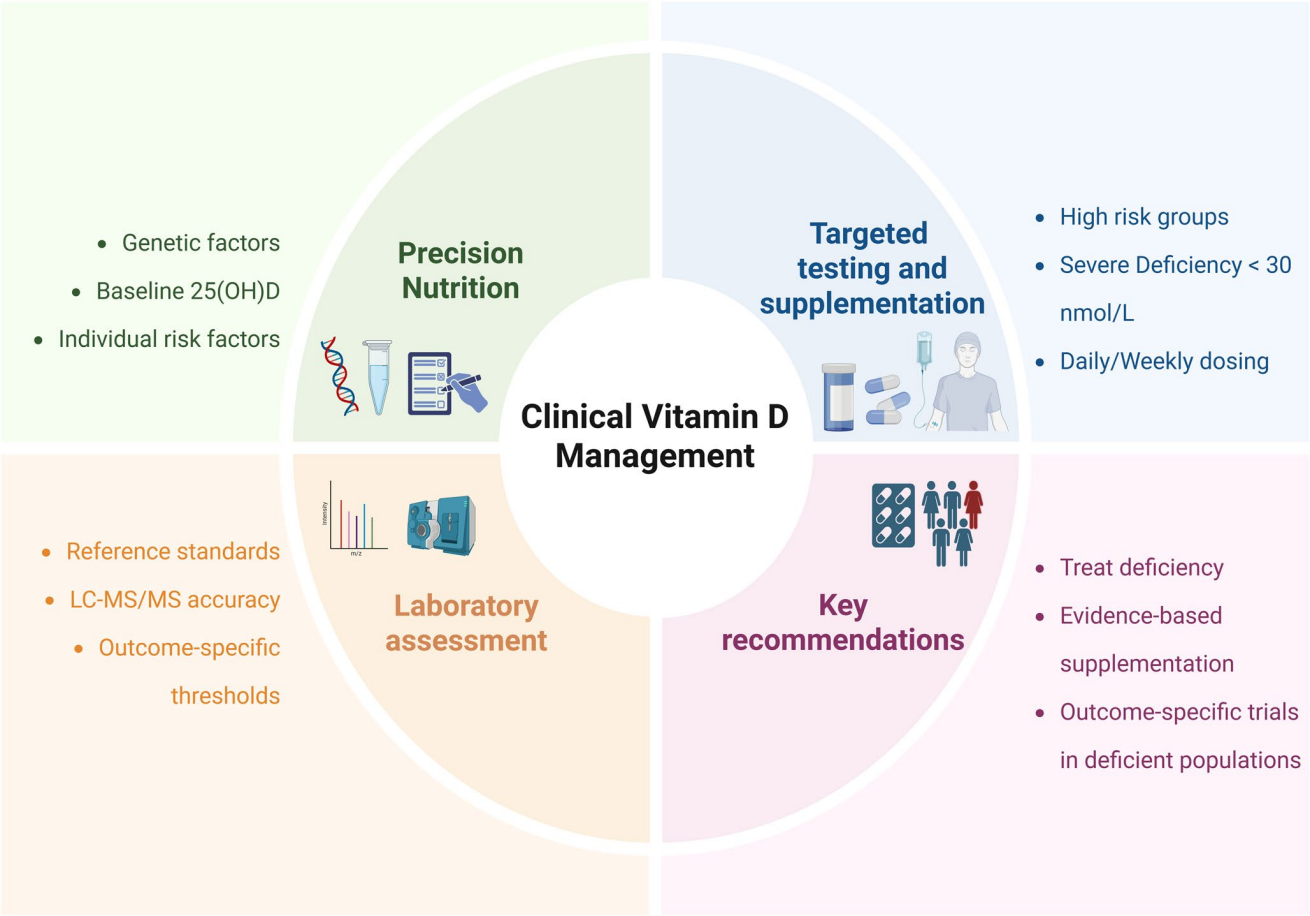
Evidence–practice gap in vitamin D management. Low circulating 25(OH)D is consistently associated with adverse outcomes, but observational findings may reflect confounding, reverse causation, or assay variability. Neutral RCTs often reflect vitamin D–replete populations, background supplementation, fixed dosing, and limited deficient-subgroup analyses. A precision approach integrates baseline status, clinical context, assay quality, and individualized risk to guide targeted testing and supplementation

Vitamin D occupies a unique position in clinical medicine, representing a nutrient with well-established skeletal functions, widespread deficiency, and persistent uncertainty regarding extraskeletal benefits. The discordance between observational associations and RCT findings reflects several methodological, biological, and analytical challenges, summarized in Fig. 3.

A key limitation has been the absence of baseline 25(OH)D eligibility criteria in most major trials, which has precluded adequately powered analyses in deficient subgroups, where benefits are most biologically plausible [5, 14].

Current evidence supports a targeted rather than universal approach to vitamin D management. Routine population screening is not recommended; instead, testing should be reserved for individuals at high risk of deficiency or when results will directly influence clinical decisions. Empiric supplementation is justified in specific groups, including children and adolescents, pregnant women, adults aged ≥ 75 years, and individuals with high-risk prediabetes, for whom RCT evidence demonstrates meaningful benefits [11, 39]. The 2024 ES guideline frames these as conditional recommendations based on varying certainty of evidence, high for mortality reduction in adults aged ≥ 75 years, moderate for prediabetes progression, and low for pregnancy-related and pediatric respiratory outcomes [5]. For skeletal and other outcomes, clinically significant effects are primarily observed in states of deficiency, although selected benefits may be population- and outcome-specific [14]. However, the precise 25(OH)D thresholds at which benefits emerge remain undefined for most outcomes, as no RCTs have been designed or powered to determine outcome-specific thresholds [5].

The key message for clinicians is threefold: (1) prevent and correct severe deficiency (< 12 ng/mL [< 30 nmol/L]), which causes unequivocal harm; (2) target supplementation to evidence-based indications rather than pursuing universal optimization; and (3) favor daily or weekly dosing over intermittent high-dose regimens, as doses > 100,000 IU may be associated with increased fracture risk (RR 1.14, 95% CI 1.02–1.27) and dosing intervals > 12 weeks may increase fall risk (RR 1.08, 95% CI 1.03–1.14) [5, 85]. Ultimately, bridging the gap between evidence and practice requires harmonized assays, outcome-specific threshold definitions, and trials designed to evaluate vitamin D in deficient populations, where its true clinical potential may yet be realized. Future research should also incorporate individualized response phenotyping, integrating genetic, metabolic, and clinical variables, to move beyond uniform population targets toward precision-guided supplementation strategies.

## Key References

- Dalamaga M, Emfietzoglou R, Petropoulou D, Kypraiou M, Kounatidis DC, Vallianou NG, Karras S, Magkos F, Karampela I. Vitamin D and Health Outcomes: State-of-the-Art Review of Triangulated Evidence and Ongoing Controversies. Curr Nutr Rep. 2026; 15(1):26. 10.1007/s13668-026-00748-2 (of high importance)
  - ○ This review synthesizes evidence from observational studies, Mendelian randomization, and RCTs to clarify vitamin D’s causal health effects. It finds strong support for vitamin D’s role in skeletal health, especially in deficiency-related conditions and fracture prevention in older or deficient groups. Extraskeletal benefits appear modest and mainly limited to those with low baseline vitamin D, while evidence is weak for cardiovascular, metabolic, obesity, and most neuropsychiatric outcomes.
- Liu D, Meng X, Tian Q, Cao W, Fan X, Wu L, Song M, Meng Q, Wang W, Wang Y. Vitamin D and Multiple Health Outcomes: An Umbrella Review of Observational Studies, Randomized Controlled Trials, and Mendelian Randomization Studies. Adv Nutr. 2022; 13(4):1044-1062. 10.1093/advances/nmab142. (of high importance)
  - ○ This is an Umbrella Review of Observational Studies, Randomized Controlled Trials, and Mendelian Randomization Studies regarding vitamin D and multiple health outcomes.
- Fang A, Zhao Y, Yang P, Zhang X, Giovannucci EL. Vitamin D and human health: evidence from Mendelian randomization studies. Eur J Epidemiol. 2024;39():467-490. 10.1007/s10654-023-01075-4. (of importance)
  - ○ Evidence from linear Mendelian randomization studies provides strong support for a causal link between vitamin D and the development of multiple sclerosis.
- Demay MB, Pittas AG, Bikle DD, Diab DL, Kiely ME, Lazaretti-Castro M, et al. Vitamin D for the Prevention of Disease: An Endocrine Society Clinical Practice Guideline. J Clin Endocrinol Metab. 2024;109(8):1907-1947. 10.1210/clinem/dgae290. (of importance)
  - ○ The Endocrine Society panel recommends empiric vitamin D supplementation for individuals aged 1–18 years, adults over 75 years, pregnant individuals, and those with high-risk prediabetes.

## Data Availability

No datasets were generated or analysed during the current study.

## Abbreviations

1,24,25(OH)_3_D: 1,24,25-trihydroxyvitamin D
1,25(OH)_2_D: 1,25-dihydroxyvitamin D (calcitriol)
24,25(OH)_2_D: 24,25-dihydroxyvitamin D
25(OH)D: 25-hydroxyvitamin D
25(OH)D_2_: 25-hydroxyvitamin D_2_
25(OH)D_3_: 25-hydroxyvitamin D_3_
3-epi-25(OH)D_3_: 3-epimer of 25-hydroxyvitamin D_3_
AACE: American Association of Clinical Endocrinologists
AME: Italian Association of Clinical Endocrinologists
BHOF: Bone Health and Osteoporosis Foundation
BMD: bone mineral density
BMI: body mass index
CDC: Centers for Disease Control and Prevention
CI: confidence interval
CKD: chronic kidney disease
CKD–MBD: chronic kidney disease–mineral and bone disorder
CLIA: chemiluminescent immunoassay
CYP: cytochrome P450
CYP2R1: cytochrome P450 2R1 (vitamin D 25-hydroxylase)
CYP3A4: cytochrome P450 3A4
CYP24A1: cytochrome P450 24-hydroxylase
CYP27A1: cytochrome P450 27A1
CYP27B1: 1α-hydroxylase
D-Health: D-Health Trial
D₂: ergocalciferol
D_3_: cholecalciferol
DEQAS: Vitamin D External Quality Assessment Scheme
DHCR7/NADSYN1: 7-dehydrocholesterol reductase/NAD synthetase 1
DRIs: Dietary Reference Intakes
ECTS: European Calcified Tissue Society
EFSA: European Food Safety Authority
ELISA: enzyme-linked immunosorbent assay
ES: Endocrine Society
EU: European Union
FAIRE-qPCR: formaldehyde-assisted isolation of regulatory elements followed by quantitative polymerase chain reaction
FGF23: fibroblast growth factor 23
GRS: genetic risk score
GWAS: Genome-wide association studies
HPLC: high-performance liquid chromatography
HR: hazard ratio
IA: immunoassay
IOM: Institute of Medicine
IU: international units
JCTLM: Joint Committee for Traceability in Laboratory Medicine
KiGGS: German Health Interview and Examination Survey for Children and Adolescents
LC–MS/MS: liquid chromatography–tandem mass spectrometry
MR: Mendelian randomization
NAM: National Academy of Medicine
NHANES: National Health and Nutrition Examination Survey
NIH: National Institutes of Health
NIST: National Institute of Standards and Technology
OR: odds ratio
PTH: parathyroid hormone
RCT: randomized controlled trial
RIA: radioimmunoassay
RMP: reference measurement procedure
RR: relative risk
SI: International System of Units
SNP: single-nucleotide polymorphism
UL: tolerable upper intake level
USPSTF: U.S. Preventive Services Task Force
UV: ultraviolet
UVB: ultraviolet B
VDBP: vitamin D–binding protein
VDR: vitamin D receptor
VDSP: Vitamin D Standardization Program
VITAL: VITamin D and OmegA-3 TriaL
VMR: vitamin D metabolite ratio
